# Gene expression responses in *Suaeda salsa* after cadmium exposure

**DOI:** 10.1186/2193-1801-2-232

**Published:** 2013-05-20

**Authors:** Ming Cong, Jiasen Lv, Xiaoli Liu, Jianmin Zhao, Huifeng Wu

**Affiliations:** Key Laboratory of Coastal Zone Environmental Processes, Yantai Institute of Coastal Zone Research(YIC), Chinese Academy of Sciences(CAS); Shandong Provincial Key Laboratory of Coastal Zone Environmental Processes, YICCAS, Yantai Shandong, 264003 P. R. China; Ocean School of Yantai University, Yantai, 264005 P. R. China; The Graduate School of Chinese Academy of Sciences, Beijing, 100049 P. R. China

**Keywords:** *Suaeda salsa*, qRT-PCR, Gene expression, Cadmium

## Abstract

Coastal line is now polluted by many kinds of sewage including heavy metals discharged by intensive human activities. Cadmium is a nonessential heavy metal for organisms and can cause many kinds of adverse effect on the organisms. *Suaeda salsa*, a pioneer halophyte in intertidal zone of the Bohai coast, was proved to have cadmium-tolerant capacity. Given that, *S. salsa* was suggested as a potential coastal bio-indicator plant for cadmium contamination in the intertidal zone. Therefore, it is essential to investigate the responsive mechanism of *S. salsa* to cadmium since few studies focus on this subject till now. In the present study, six genes were selected to investigate the variation profiles of mRNA expression by fluorescent real-time quantitative PCR, including those involved in *myo-inositol* synthesis, redox reaction, salt-tolerant reaction. Results showed that cadmium exposure significantly modulate the mRNA expressions of MIPS, Nhx1, CAT2, GST, Prx Q genes. It suggested that cadmium exposure exerted an oxidative stress on *S. salsa*, disturbed Na^+^ homeostasis across membranes and interfered with the metabolism of inositol. In addition, CAT2 gene could be used as a gene marker in *S. salsa* to indicate cadmium pollution.

## Introduction

Coastal line is now experiencing increasingly pollution pressure which comes from highly intensive human activities. Heavy metals are important contaminants from factory sewage. Cadmium, an abundant and nonessential heavy metal, can be released into coastal environment through mining, metal refining (Otte et al. 1993). Cadmium can cause adverse effects on the plants in many respects, such as disturbance in metabolism (Poschenrieder *et al.*^1989^), secondary water stress (Poschenrieder *et al.*^1989^, Nedjimi and Daoud 2009), oxidative stress (Gill and Tuteja 2010). Since heavy metal is a kind of persistent pollution for the environment, it is necessary to indicate their toxicological effects in the coastal area by a kind of immobile organism, such as plants.

*Suaeda salsa* is a native halophyte in the Bohai coast, and can grow in the intertidal zone where soil salt reaches up to 3%. It has been reported that halophytes have more efficient abilities over non-halophytic plants, such as a more efficient antioxidant system (Zhu *et al.*^2004^), synthesizing more osmo-protectants in order to keep a favorable water potential gradient and to protect normal cellular function (Lefèvre *et al.*^2009^). These advantages offer the halophytes to share similar physiological mechanisms with heavy metal-tolerant plants (Thomas *et al.*^1998^, Przymusinski *et al.*^2004^). Recently, quite a few of papers have reported that *S. salsa* can tolerate several pollutants, including petroleum hydrocarbons, heavy metals (Zhu *et al.*^2005^, Xu *et al.*^2007^, Liu and Xu 2008). As a pioneer halophyte, *S. salsa* was suggested to be a potential plant in pollution indication around heavy metal-contaminated coast.

But few studies focused on the underlying mechanism of *S. salsa* to the pollutants. Our previous studies demonstrated that cadmium of environmentally relevant concentrations (up to 50 μg L^-1^) had no significant effect on the total weight of *S. salsa*, while the cadmium contents in the Cd-exposed *S. salsa* were significantly higher than that of the control group in a dose-dependent manner ( Liu *et al.*^2011^). On the other hand, significant alteration in basic metabolisms, and antioxidant enzyme activities (including catalase and glutathione S-transferases) were also recorded in *S. salsa* (Liu *et al.*^2011^). It can be postulated that the correlated gene expression profile of *S. salsa* was also changed significantly, because variation of gene expression preceded that of the metabolism (Bennett *et al.*^1993^). Therefore, investigation on the mRNA variation profiles of some related genes would provide an early and sensitive alarm on heavy metal contamination.

In this study, several stress-related genes were selected to be evaluated by fluorescent real-time quantitative PCR. *Myo*-inositol-1-phosphate synthase (MIPS) is the rate-limiting enzyme in biosynthesis of *myo*-inositol which is central in many biochemical and physiological processes including growth and development, oxidation, cellular protection (Majumder et al. 1997, Murata et al. 2003). Na^+^/H^+^ antiporter (Nhx) plays a crucial function in exchanging of Na^+^ for H^+^ across membranes, and is important for the plants in salt tolerance by maintaining homeostasis of cellular ion (Apse *et al.*^1999^, Horie and Schroede 2004, Xue *et al.*^2004^). Nhx gene was reported to have been cloned from *S. salsa* and then transferred into rice, which had a higher resistance to salt stress (Zhao *et al.*^2006^). However, no available studies described the response of Nhx gene to cadmium stress yet.

Additionally, cadmium can stimulate production of reactive oxygen species (ROS) which brings damage to a lot of important molecules, such as proteins, lipid, DNA (Stohs and Bagchi 1994, Foyer *et al.*^1997^). Several antioxidant enzymes including CAT and GST were reported to change significantly in response to cadmium exposure in *S. salsa* ( Liu *et al.*^2011^). Considering the results in our previous study, the expression levels of a series of antioxidant enzyme genes, including CAT, GST and Prx Q genes were also examined in the present study to investigate their involvement during the oxidative pressure from cadmium pollution.

## Materials and methods

### Plant culture and treatments

Seeds of *S. salsa* were collected from the Yellow River Delta in November, 2010 and stored at 4°C. The seeds were sterilized by immersion in 0.5% HgCl_2_ for 10 min, and then washed twice in sterilized distilled water. One hundred of plump seeds were sown in sterilized sands in 4 flowerpots with a diameter of 20 cm (*n* = 25). Plants in each pot were used as an independent group to receive different treatment. Of the four pots, one pot was used as control group and the other three were used for Cd-treated groups. All of them were irrigated with Hoagland’s nutrient solution at room temperature first. After germination, seeds were cultivated in a plant incubator with 28 ± 4°C, photoperiod 12 h light/12 h darkness, relative humidity 70% and photo-synthetically active radiation of 600 μmol m^-2^ s^-1^. After cultivation for 4 weeks, plantlets of the Cd-exposed groups were irrigated with Hoagland’s nutrient solution containing gradient concentrations (2, 10 and 50 μg L^-1^) of Cd, respectively, and the highest concentration were environmentally relevant to the real situation of Cd pollution in the seawater along the intertidal zone of the Bohai Sea (Zhang 2001). Accordingly, the Cd concentrations of 2, 10 and 50 μg/L were considered as low (L), moderate (M) and high (H) concentrations, respectively. Considering the biomass from different tissues, leaves of *S. salsa* were collected as samples to extract RNA. After exposure to Cd for 1, 2 and 3 weeks, five plantlets in each group were used to collect leaves, and leaves from one plantlet were used as one sample. All of the samples were flash-frozen in liquid nitrogen before storing at −80°C prior to RNA extraction.

### RNA extraction and gene quantification

Total RNA from leaves was isolated following the manufacturer’s directions (Invitrogen), and the first-strand cDNA synthesis was carried out according to M-MLV RT Usage information (Promega) using oligo (dT)-adaptor (5′-CTCGAGATCGATGCGGCCGCT_17_-3′) as primer and the DNase I-treated (Promega) total RNA as template.

Gene-specific primers for *Nhx*, *CAT1*, *CAT2*, *GST*, *Prx Q*, *MIPS* and the internal control *β-actin* were used to amplify amplicons specific for *S. salsa*. The sequences of primers and the length of amplicons were shown in Table 1. The fluorescent real-time quantitative PCR amplifications were carried out in triplicate in a total volume of 20.0 μl containing 10.0 μl of 2 × SYBR Premix Ex Taq™ (TaKaRa), 0.4 μl of 50 × ROX Reference DYE ІІ, 4.8 μl DEPC-treated H_2_O, 0.4 μl of each primer, 4.0 μl of 1:20 diluted cDNA. The fluorescent real-time quantitative PCR program was as following: 50°C for 2 min and 95°C for 10 min, followed by 40 cycles of 94°C for 15 s, 58°C for 45 s, 72°C for 30 s. Dissociation curve analysis of amplification products was performed at the end of each PCR to confirm that only one PCR product was amplified and detected. The final PCR products were purified and sent to be sequenced, and then aligned with the sequence information deposited in NCBI to verify the right amplification.

**Table 1:** **Specific primers used in RT-PCR**

Gene		Primers (5′-3′)	Length of amplicon (bp)
Actin (BE231408)	Forward	ATCCGCAAAGATTACATACCATA	254
	Reverse	TTGTTCACCGAAAGTGCTTCT	
Prx Q (AY373447)	Forward	GCACAAGGCATTCAAACAGAAG	160
	Reverse	AACAAGTCGGACAACACCGT	
GST (BE859255)	Forward	TCCGCAAAGATTACATACCATA	293
	Reverse	GTGGATCTCCAAGGGCGAGTA	
MIPS (BE644574)	Forward	CTTCTTCGTTTTCCCCTCTT	155
	Reverse	AGCCTTTGCGATTCTCGT	
CAT1 (AF390210)	Forward	ACTTCCCATCAAGATACGACCCT	256
	Reverse	GATTTGTCAGCCTGAGACCAGTA	
CAT2 (AY046530)	Forward	GGACTTTCGCCTATGCTGAT	265
	Reverse	CCTGGCTCCTTGAAGTTATTC	
Nhx1 (AY261806)	Forward	TGTTGCTGTGAGTTCCATATT	208
	Reverse	TGTGTGCCCTGACCTTGA	

### Statistical analysis

After the PCR program, data were analyzed with the ABI 7500 SDS software (Applied Biosystems). To maintain consistency, the baseline was set automatically by the software. The comparative CT method (2^-∆∆CT^ method) was used to analyze the expression levels of the genes (Livak and Schmittgen 2001). One-way ANOVA followed by least significant difference (LSD) test was performed to analyze statistical significance by using SPSS (Version 16.0) among the data from the control (C), low (L), moderate (M) and high (H) groups at each time point. All the data were expressed as mean ± standard deviation (S.D.) (n = 5).

## Results

**Figure 1:**
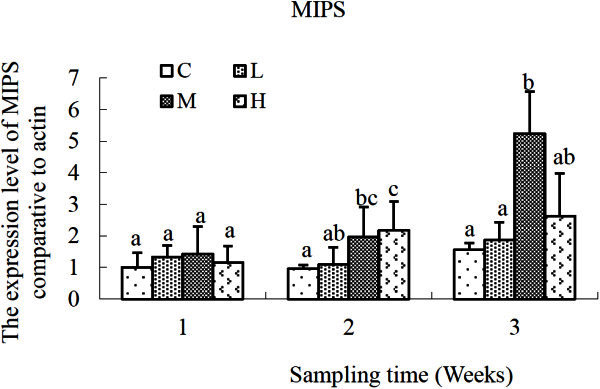
**Relative expression levels of MIPS gene after exposure to different concentrations of cadmium.** C, control; L, 2 μg L^-1^; M, 10 μg L^-1^; H, 50 μg L^-1^. β-actin gene was used as an internal reference to evaluate the comparative expression level of MIPS. Different letters above each column denoted significant difference between them (*p* <0.05).

At the first sampling time, MIPS didn’t exhibit significantly difference in *S. salsa* exposed by either low (L, 2 μg L^-1^), moderate (M, 10 μg L^-1^) or high (H, 50 μg L^-1^) concentration of cadmium (Figure 1). After exposure for two weeks, significant up-regulations of MIPS expression were observed in *S. salsa* of M and H groups (*P* < 0.05, *P* < 0.01) compared with that of the control group. Although slight increment was observed in the plants of L group, there was no significant difference compared with the control (*P* > 0.05). Neither was that between the L and M groups (*P* > 0.05). Three-weeks later, only the moderate concentration (10 μg L^-1^) of cadmium exerted remarkably significant increment (2.7-fold, *P* < 0.01) on MIPS transcript expression compared with that of the control group. However, low and high concentrations of cadmium didn’t affect MIPS transcription significantly (*P* > 0.05).

**Figure 2:**
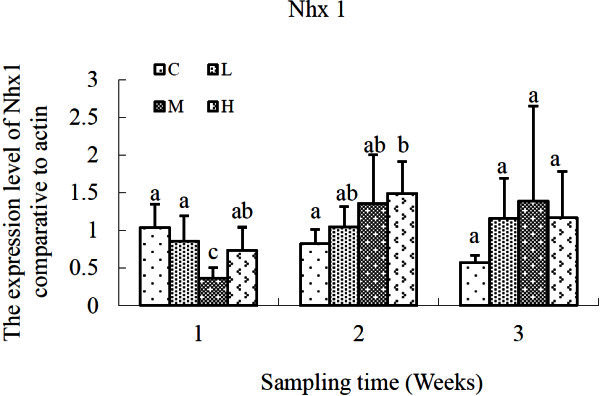
**Relative expression levels of NHX1 gene after exposure to different concentrations of cadmium.** C, control; L, 2 μg L^-1^; M, 10 μg L^-1^; H, 50 μg L^-1^. β-actin gene was used as an internal reference to evaluate the comparative expression level of MIPS. Different letters above each column denoted significant difference between them (*p* <0.05).

The variation profile of Nhx1 mRNA expression was shown in Figure 2. No consistent results were discovered with regard to cadmium levels or time duration. At the first sampling time (1 week), down-regulation of Nhx1 expression were detected in *S. salsa* of M and H groups, but only those in M group exhibited a significant decrease compared with that of the control group (*P* < 0.05). As time elapsed, increments of Nhx1 gene expression occurred in all of the cadmium-exposed groups at the second sampling time (2 weeks). But only *S. salsa* in H group expressed a significantly higher (*P* < 0.05) level compared with that of the control group. At the end of the third week, no significant difference of Nhx1 expression was detected in the three Cd-exposed groups.

**Figure 3:**
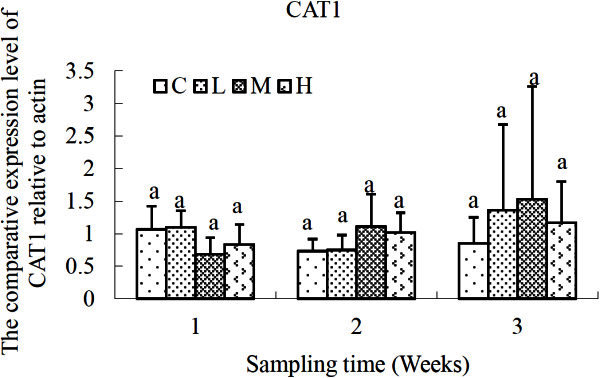
**Relative expression levels of CAT1 gene after exposure to different concentrations of cadmium.** C, control; L, 2 μg L^-1^; M, 10 μg L^-1^; H, 50 μg L^-1^. β-actin gene was used as an internal reference to evaluate the comparative expression level of MIPS. Different letters above each column denoted significant difference between them (*p* <0.05).

**Figure 4:**
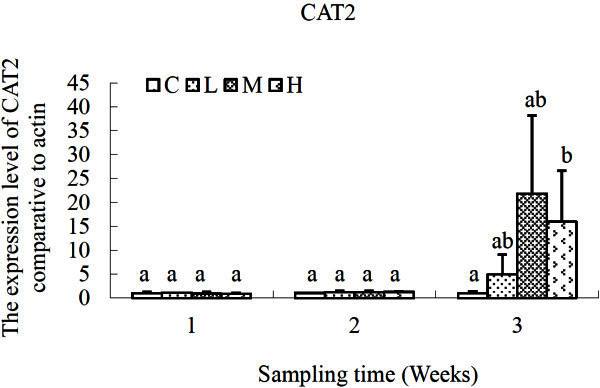
**Relative expression levels of CAT2 gene after exposure to different concentrations of cadmium.** C, control; L, 2 μg L^-1^; M, 10 μg L^-1^; H, 50 μg L^-1^. β-actin gene was used as an internal reference to evaluate the comparative expression level of MIPS. Different letters above each column denoted significant difference between them (*p* <0.05).

The changing profiles of CAT (CAT1, CAT2) genes were different as shown in Figures 3 and 4. Although there was variation in the expression profile of CAT1 gene, no significant difference was recorded over time or exposure levels. For CAT2 gene, significant increment of CAT2 expression was detected in *S. salsa* of H group in comparison with that of the control group after exposure for 3 weeks (*P* < 0.05). And there was no significant difference in L and M groups compared with the control.

**Figure 5:**
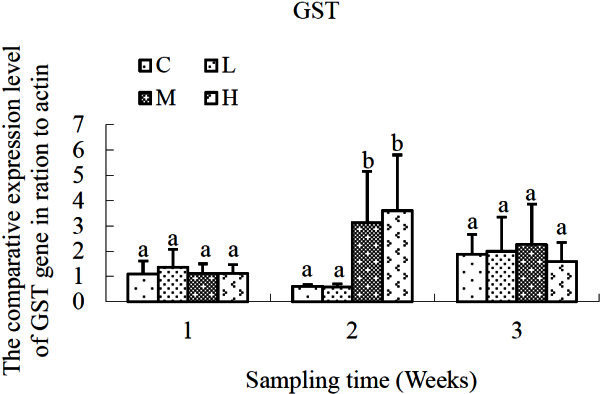
**Relative expression levels of GST gene after exposure to different concentrations of cadmium.** C, control; L, 2 μg L^-1^; M, 10 μg L^-1^; H, 50 μg L^-1^. β-actin gene was used as an internal reference to evaluate the comparative expression level of MIPS. Different letters above each column denoted significant difference between them (*p* <0.05).

As for GST gene (Figure 5), there was no significant difference in *S. salsa* exposed to different concentrations of cadmium compared with that of the control group after 1-week-exposure. But significant higher transcriptional levels of GST gene were detected in *S. salsa* of M and H groups (*P* < 0.05) after cadmium exposure for 2 weeks. And no significant difference was detected in each cadmium-exposed group in comparison with that of the control group at the third sampling time-point.

**Figure 6:**
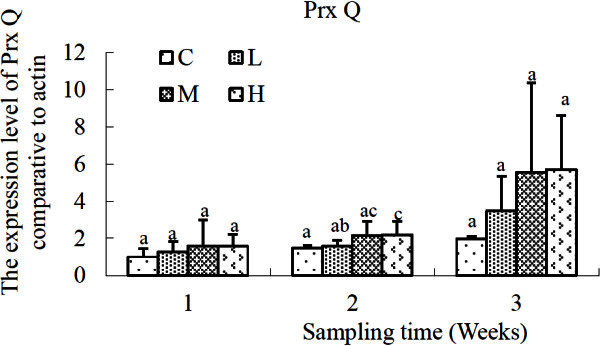
**Relative expression levels of PrxQ gene after exposure to different concentrations of cadmium.** C, control; L, 2 μg L^-1^; M, 10 μg L^-1^; H, 50 μg L^-1^. β-actin gene was used as an internal reference to evaluate the comparative expression level of MIPS. Different letters above each column denoted significant difference between them (*p* <0.05).

Variation trend of Prx Q gene was shown in Figure 6. No significant difference occurred in the expression level of Prx Q transcripts among the four groups at the first and third sampling-times. However, higher expression level of Prx Q gene was observed in *S. salsa* exposed to high (50 μg L^-1^) concentrations of cadmium at the second sampling time compared with that of the control (*P* < 0.01) and that of the L group (*P* < 0.05).

## Discussion

Previous study suggested that *S. salsa* has cadmium-tolerant capability. In the present study, some important genes which might participate in detoxifying response to cadmium exposure were analyzed in order to better understand the underlying mechanism and screen bio-indicators for environmental pollution in future.

Moderate (10 μg L^-1^) and high (50 μg L^-1^) concentrations of cadmium could effectively up-regulate MIPS transcripts at the second sampling time-point. And 10 μg L^-1^ of cadmium exhibited cumulative effects of time duration on enhancement of MIPS transcription. Interestingly, as time elapsed, 50 μg L^-1^ of cadmium had no more significant effect on gene transcription of MIPS at the third sampling time-point. It maybe imply an adaptive cadmium-tolerance mechanism emerging in *S. salsa* to avoid extra damage, since strong induction of some stress-irritable factors could trade off other important functions of organisms (Brulle *et al.*^2007^). Alternatively, other heavy metal chelators such as phytochelatin, metallothionein-like protein would be induced significantly by a higher concentration (50 μg L^-1^) of cadmium exposure (Tran and Popova 2013). It would be further investigated in the future study. Given that MIPS is a pivotal enzyme to catalyze *myo*-inositol which metabolic products (such as myo-inositol hexakisphosphate) impact uptake and translocation of heavy metals (Loewus and Murthy 2000), the significant up-regulation of MIPS transcripts suggested that 10 μg L^-1^ and 50 μg L^-1^ of cadmium affected the metabolic pathway of *myo*-inositol and the detoxification function by its downstream products probably.

Na^+^/H^+^ antiporter (Nhx1) is an important membrane protein responsible for pumping Na^+^ into vacuole in order to reduce Na^+^ toxicity and alleviate the adverse effect of salt stress (Zhang *et al.*^2008^). Investigation of Nhx1 gene mRNA expression in the present study was helpful to find out whether cadmium exposure affected salt-tolerance ability of *S. salsa*. In our study, a decreased expression level of Nhx1 gene was detected in *S. salsa* exposed to medium concentration (10 μg L^-1^) of cadmium at the first sampling time. It implied that one-week exposure of 10 μg L^-1^ of cadmium impaired the normal homeostasis of Na^+^ around the membrane, and decreased the salt-tolerance ability of *S. salsa*. But as time elapsed, a normal balance was re-gained in the M group at the second sampling time-point. And significant enhancement of Nhx1 transcripts occurred in response to high concentration (50 μg L^-1^) of cadmium. Previous transgenic studies revealed that overexpression of Nhx gene could significantly enhance the plants’ salt-tolerance ability (Qian and Zhang 2007). Therefore, the result suggested that two-week exposure to 50 μg L^-1^ of cadmium induced higher salt-tolerance ability in *S. salsa*. But this kind of effect disappeared at the third sampling time-point. It was probably another presentation of adaptive response of *S. salsa* to continuous cadmium exposure.

Taken the antioxidant enzymes altogether, CAT, GST and Prx Q are all essential enzymes in ROS metabolism. In plants, CAT plays important roles in the metabolism of peroxide by converting hydrogen peroxide into water and signal transduction in many defense reactions (Kendall *et al.*^1983^, Polidoros *et al.*^2001^). Presently, two kinds of CAT genes were analyzed. CAT1 gene had no significant variation in expression level during three weeks at any dose of cadmium. However, exposure to high concentration (50 μg L^-1^) of cadmium enhanced the expression of CAT2 gene significantly. It implied that CAT2 gene was more responsive than CAT1 gene towards cadmium exposure. Accordingly, CAT2 gene might play dominating role in the increased CAT enzyme activity in *S. salsa* towards cadmium exposure.

As for another antioxidant enzyme gene, significant increments of GST expression were recorded in *S. salsa* exposed to both moderate and high concentrations of cadmium after two-week exposure. Since GST is responsible for the conjugation of GSH with other electrophilic substance from aromatic hydrocarbons, heavy metals and so on (Hossain et al. 2012), an increased GST activity toward cadmium exposure might imply that more GSHs were involved in the formation of Cd-binding complexes to alleviate the toxicological effects of cadmium. Similarly an increased GST activity was reported in soybean in response to cadmium exposure (Yang et al. 2012). In addition to GST and GSH, phytochelatins, heavy metal transporter proteins and other small organic acid all played important detoxifying functions to defend against free ion of heavy metals inside the cells (Tran and Popova 2013). They acted together to bind the heavy metals in location or transport them from root to shoot. Increased GST activity at 2-week-time and decreased at 3-week-time probably suggested that GST was mainly played detoxifying roles during the first 2-weeks. And other detoxifying factors would probably participate in the defense response to cadmium 3-weeks later.

The higher expression levels of CAT and GST genes toward cadmium exposure were in accordance with our previous results that the enzyme activities of CAT and GST were significantly higher in cadmium-stressed *S. salsa* (Liu et al. 2011). But there was not good correlation in up-regulations of enzyme activities (including CAT and GST) and the corresponding mRNA expression levels in view of time. It probably because certain isoenzyme genes of one enzyme family were investigated during qRT-PCR study, but all of the same enzyme-family members were measured in enzyme activity assay. Similar phenomena were found in other antioxidant enzymes (Wu et al. 2012).

Prx Q is an important cysteine-containing antioxidant enzyme and plays central roles in many biotic and abiotic defense reactions (Navrot *et al.*^2006^). High (50 μg L^-1^) concentration of cadmium caused significant up-regulation in the expression level of *Prx Q* transcripts, followed by a reduction to the control level at the third sampling time-point. It was similar to the expression profile of GST gene. And an adaptive response might exist in the gene expression of GST and Prx Q to the continuous cadmium-exposure.

In conclusion, MIPS, Nhx1, CAT2, GST, Prx Q genes except for CAT1 were all involved in the defense reaction of *S. salsa* towards cadmium exposure. Some of them (Nhx1, GST and Prx Q) exhibited significant increments in transcript expressions at the second sampling time-points (two weeks). It suggested that Nhx1, GST and Prx Q genes might play defense functions during the first two weeks after cadmium exposure. 10 μg L^-1^ of cadmium exhibited cumulative effects of time duration on enhancement of MIPS transcription. Cadmium exposure could affect the synthesis of *myo*-inositol and probably its downstream products which played important detoxification function in cadmium uptake and translocation. Homeostasis of Na^+^ was also influenced by cadmium exposure but exhibited dose–response over time. In addition, CAT2 gene was potentially suitable to indicate cadmium pollution as a biomarker.

## Abbreviations

S. salsa: *Suaeda salsa*
Cd: Cadmium
CAT: Catalase
GST: Glutathione S-transferases
MIPS: *Myo*-inositol-1-phosphate synthase
Nhx: Na^+^/H^+^ antiporter
ROS: Reactive oxygen species
Prx Q: Peroxiredoxin Q.
